# The effect of three hemostatic agents on early bone healing in an animal model

**DOI:** 10.1186/1471-2482-10-37

**Published:** 2010-12-17

**Authors:** Jonathan K Armstrong, Bo Han, Kenrick Kuwahara, Zhi Yang, Clara E Magyar, Sarah M Dry, Elisa Atti, Sotirios Tetradis, Timothy C Fisher

**Affiliations:** 1 Department of Diagnostic and Surgical Sciences, UCLA School of Dentistry, Los Angeles, CA 90095 USA; 2 Ceremed Inc., 3643 Lenawee Avenue, Los Angeles, CA 90016 USA; 3 Department of Surgery, Keck School of Medicine, University of Southern California, Los Angeles, CA 90033 USA; 4 Department of Pathology and Laboratory Medicine, The David Geffen School of Medicine at UCLA, Los Angeles, CA 90095 USA

## Abstract

**Background:**

Resorbable bone hemostasis materials, oxidized regenerated cellulose (ORC) and microfibrillar collagen (MFC), remain at the site of application for up to 8 weeks and may impair osteogenesis. Our experimental study compared the effect of a water-soluble alkylene oxide copolymer (AOC) to ORC and MFC versus no hemostatic material on early bone healing.

**Methods:**

Two circular 2.7 mm non-critical defects were made in each tibia of 12 rabbits. Sufficient AOC, ORC or MFC was applied to achieve hemostasis, and effectiveness recorded. An autologous blood clot was applied to control defects. Rabbits were sacrificed at 17 days, tibiae excised and fixed. Bone healing was quantitatively measured by micro-computed tomography (micro-CT) expressed as fractional bone volume, and qualitatively assessed by histological examination of decalcified sections.

**Results:**

Hemostasis was immediate after application of MFC and AOC, after 1-2 minutes with ORC, and >5 minutes for control. At 17 days post-surgery, micro-CT analysis showed near-complete healing in control and AOC groups, partial healing in the ORC group and minimal healing in the MFC group. Fractional bone volume was 8 fold greater in the control and AOC groups than in the MFC group (0.42 ± 0.06, 0.40 ± 0.03 vs 0.05 ± 0.01, *P *< 0.001) and over 1.5-fold greater than in the ORC group (0.25 ± 0.03, *P *< 0.05). By histology, MFC remained at the application site with minimal healing at the defect margins and early fibrotic tissue within the defect. ORC-treated defects showed partial healing but with early fibrotic tissue in the marrow space. Conversely, control and AOC-treated defects demonstrated newly formed woven bone rich in cellular activity with no evidence of AOC remaining at the application site.

**Conclusions:**

Early healing appeared to be impaired by the presence of MFC and impeded by the presence of ORC. In contrast, AOC did not inhibit bone healing and suggest that AOC may be a better bone hemostatic material for procedures where bony fusion is critical and immediate hemostasis required.

## Background

Choosing an effective bone hemostasis material poses a unique challenge. The surgeon must weigh the efficacy, immediacy and ease of use of the material against potential unintended consequences associated with the material such as adverse effects on bone healing. Commonly used hemostatic agents encompass nonresorbable materials such as softened beeswax and resorbable materials manufactured from plants (e.g., cellulose), animal products (e.g., collagen), or synthetic polymers (e.g., alkylene oxide copolymers) [1]. All topical hemostatic agents have distinct advantages and disadvantages and a recent comprehensive review by Achneck and colleagues [1] provides the surgeon with a selection guide based on the agent's mechanism of action, efficacy and potential adverse effects.

Bonewax is composed primarily of beeswax, a hydrophobic and non-resorbable material. Bonewax remains at the implantation site indefinitely and is well known to inhibit bone growth as demonstrated in numerous animal [2-8] and clinical studies [9-13]. Although bone wax is effective in achieving immediate bone hemostasis by means of tamponade, clinicians are increasingly looking toward bone hemostatic agents that either resorb or dissolve from the site of application [1,14].

Two commonly used resorbable materials are oxidized regenerated cellulose (ORC), composed of polyanhydroglucuronic acid derived from wood pulp [15,16], and microfibrillar collagen (MFC), an animal product derived from purified bovine hide corium [17,18]. Both materials are actively degraded *in vivo *and are reported to be removed from the site of application within 6 weeks to still being present after 1 year [19,20] for ORC or within 45 to 90 days [3,21] for MFC.

Alkylene oxide copolymer (AOC) is a synthetic bone hemostasis material composed of water-soluble copolymers [14]. AOC is a hydrophilic waxy material that sticks well to wet surfaces, which makes it well-suited for bone hemostasis. Alkylene oxide copolymers have a long history of use in the medical and pharmaceutical fields [22-24]; they are biocompatible, chemically inert, non-metabolizable and are eliminated from the body unchanged primarily via renal excretion [25,26]. AOC dissolves from the site of application within 48 hours [8,24].

Resorbable bone hemostasis materials are often selected because of their perceived lack of interference with bone healing. Previous studies of bone healing in the presence of hemostatic agents have produced conflicting results, with conclusions often based on histological observations rather than quantitative measurement of bone growth [16].

This paper compares the hemostatic properties and effects on bone healing of AOC, ORC and MFC. The products ORC and MFC were selected because they are often used in surgery for bone hemostasis, and presumably it is assumed that ORC and MFC do not significantly interfere with bone healing because they are resorbable. However, as AOC dissolves from the site of application within 2 days compared to weeks/months for ORC and MFC, we hypothesized that AOC would permit faster bone healing than other resorbable hemostatic agents, and designed the current study to test this hypothesis. The animal model was designed to evaluate the effects of these agents on early healing of a non-critical size bone defect. Both bone quantity and quality were evaluated by micro-CT and histology.

The objective of this study is to provide quantitative data on early bone healing following application of resorbable bone hemostasis materials with an aim to better assist the surgeon in selection of an effective bone hemostatic material for procedures where bony fusion is critical and early healing is desired.

## Methods

### Materials

Three resorbable hemostatic agents were used for this study: Alkylene oxide copolymer ("AOC", Ostene^®^, Ceremed Inc., Los Angeles, California); oxidized regenerated cellulose ("ORC", Surgicel^®^, Ethicon Inc., Somerville, New Jersey); and microfibrillar bovine collagen ("MFC", Avitene^®^, Davol Inc., Warwick, Rhode Island).

### Rabbit Tibia Defect Model

The animal protocol was approved by the Institutional Animal Care and Use Committee at the University of Southern California, in conformity with the NIH-guidelines for the care and use of laboratory animals (DHHS publication No. [NIH] 85-23 revised 1996).

Micro-computed tomography (micro-CT) analyses of non critical-size bilateral bone defects were performed based on previous studies [6,27]. Twelve 2.5 kg male New Zealand White rabbits were used for this study, randomized into four treatment groups (2 defects per tibia, 4 defects per animal, 12 defects per group). Rabbits were anesthetized with a subcutaneous injection of ketamine/xylazine (90/10 mg/kg) and anesthesia maintained with 1% isoflurane. Surgery was performed using standard aseptic techniques. Tibiae were exposed and two 2.7 mm circular defects approximately 1 cm from the proximal extremity were created in each tibia with a high torque, low speed trephine drill under constant saline irrigation. Sufficient hemostatic material was applied to the defect to achieve hemostasis. For control rabbits (i.e., no hemostatic material applied), autologous blood was drawn from the ear artery prior to the surgical procedure into a 1 mL polypropylene syringe without any anticoagulant, thrombin was added to blood sample to promote clot formation and the formed clot was applied to the defect. The same hemostatic material was used for each of the two circular defects per tibia; different treatments were used for left and right tibiae per animal. The wounds were then closed with non-resorbable monofilament sutures. All animals were given a subcutaneous injection of buprenorphine (0.05 mg/kg) immediately after surgery and every 12 hours for 2 days post-surgery. Rabbits were monitored daily for the first five days and every two days thereafter for any adverse events during the 17 days post-surgical housing.

At 17 days post-surgery, rabbits were sacrificed using an intravenous overdose of pentobarbital and the tibiae were excised. The tibiae were placed in neutral buffered formalin for 48 h, washed in water and then trimmed to 2 - 3 cm sections (approximately to 0.5 cm above and below the defect site). After trimming, the tibiae were stored in 70% ethanol.

### Evaluation of Bleeding or Hemostasis

After application of a hemostatic material (or pre-formed blood clot for control defects), observations were recorded for a minimum period of 5 minutes, including time to achieve hemostasis and effectiveness of hemostasis over the observation period.

### Micro-CT Analysis

Excised, fixed tibiae were immobilized in a scanning tube using a 30% (w/w) optically-clear solution of Pluronic F108 as described previously [28]. In brief, a scanning tube was half-filled with cold F108 solution (below the gelation temperature of 17°C) and allowed to gel at room temperature. Tibiae were inserted into the gel in the desired orientation, after which the tube was then filled up with cold F108 solution and allowed to gel.

Tibiae were scanned in F108 gel using a Scanco40 scanner (micro-CT-40 Scanco Medical, Bassersdorf, Switzerland) with a voxel isotropic resolution of 15 μm and an X-ray energy of 55 KVp and 72 μA.

Two tibiae were accommodated in the scanning tube for each scan. The scan time was about 3 h and accounted for 1000 projections/180°, 200 ms integration time, and 2048 CCD detector array. A calibration phantom of hydroxyapatite was used. Segmented images were obtained using a Gaussian filter sigma = 1.2 and support = 1. Threshold for the image binarization was set as 25% of the maximal grayscale for all the scans. Volumetric analysis was performed by obtaining volumes of interest, using the dedicated Scanco software to include only the mineralized tissue formed inward during healing and separated from the rest of the tibia (i.e., 1.35 mm radius from center of defect).

The architectural parameters obtained from the binarized volumes of interest were:

total volume (TV, mm^3^), bone volume (BV, mm^3^), and bone volume fraction (BV/TV). Bone mineral density (BMD, mgmL^-1^) of mineralized tissue within the volume of interest was also measured.

TV is the volume of the whole defect (2.7 mm diameter), BV represents the volume of the mineralized tissue formed during healing and BV/TV is the relative bone volume which normalizes the volume of the mineralized tissue formed during healing by taking into account differences in thickness of the specimen. BMD is the density of mineralized tissue within the volume of interest, and is an indicator of the quality of mineralized tissue.

### Histology

Following micro-CT analysis, fixed tibiae were decalcified by immersion in excess 12 wt% hydrochloric acid (Decalcifier II^®^, Surgipath Medical Industries Inc., Richmond IL) for approximately 5 days, wax embedded, and 5 μm sections of decalcified tibiae were cut approximately 200 μm from the outer edge of the defects and near the center of the defects (approximately 1.4 mm from the outer edge).

Serial sections were stained with hemotoxylin and eosin (H&E) and with modified Masson's trichrome-elastic van Gieson (EVG) for collagen visualization. With the trichrome-EVG stain, mineralized bone is seen as red, and Type 1 collagen stains blue. Type 1 collagen is present both in fibrous or scar tissue (composed of fibrocytes or fibroblasts in a Type 1 collagen matrix) and in newly formed woven bone. The woven bone typically stains darker blue than fibrous tissue, and can also be recognized by its location and architecture.

Adjacent sections were stained with H&E vs. trichrome-EVG for appropriate comparison between images (5 μm distance between stain pairings).

Photomicrographs of sections were obtained using a ScanScope XT System (Aperio Technologies Inc, Vista, CA) at x20 magnification. Sections were examined by an expert who was blinded to the three treatment groups for mineralized bone quality, cellular activity including osteoblasts and osteoid, as well as any evidence of fibrotic tissue formation within the defect site.

### Statistics

Architectural micro-CT values (BV, TV, BV/TV) were reported as means and standard errors. BV/TV results were compared by the Tukey's Multiple Comparison Test (Prism; GraphPad Software Inc., San Diego, CA). A *P *value of <0.05 was considered statistically significant.

## Results

Hemostasis was achieved immediately after application of AOC and MFC, and was maintained for the five minute observation period. No differences were noted between AOC or MFC. Hemostasis was achieved between 1 and 2 minutes after application of ORC, and was also maintained for the five minute observation period. A period of greater than 5 minutes was required for control defects to achieve hemostasis (from 10 to about 30 minutes). Post-surgery, all rabbits were healthy with good appetites and showed signs of good healing with no evidence of complications (e.g., hematoma, infection).

**Figure 1:**
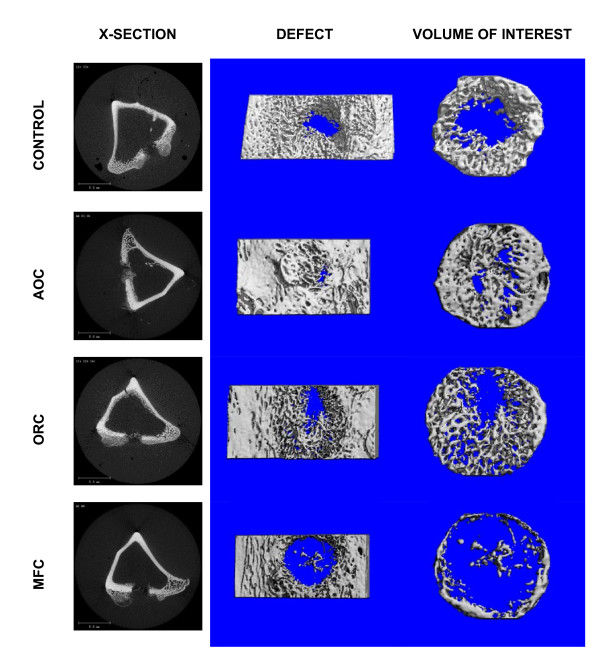
**Representative micro-CT images of excised rabbit tibiae at 17 days post-surgery**. ***Left column***: Cross-section views through the center of the defect showing newly formed bone as slightly opaque in untreated (control), alkylene oxide copolymer- and oxidized regenerated cellulose-treated defects, and an absence of opacity in microfibrillar collagen-treated defects (bar = 5.0 mm). ***Center and right columns***: micro-CT generated binarized images of mineralized tissue of the defect area (center) and area analyzed within the defect (right). AOC-treated and untreated (control) defects show substantial, well developed mineralized tissue, ORC-treated defect shows a more diffuse structure of mineralized tissue, and minimal mineralized tissue is observed within the MFC-treated defect.

**Figure 2:**
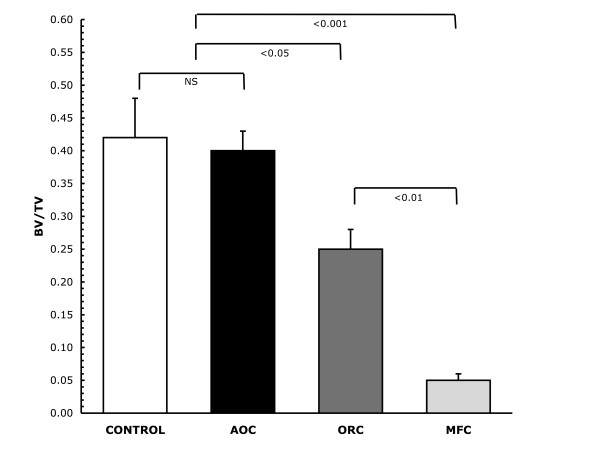
**Bone mineralization at 17 days post-surgery**. Mean values for micro-CT derived fractional bone volume (BV/TV) showing an 8 fold greater BV/TV value for untreated (control) and alkylene oxide copolymer-versus microfibrillar collagen-treated defects (0.42 ± 0.06, 0.40 ± 0.03 vs 0.05 ± 0.01 respectively, *P *< 0.001) and over 1.5 fold greater BV/TV value versus oxidized regenerated cellulose-treated defects (0.25 ± 0.03, *P *< 0.05). Number of defects per group = 12, *P *values determined from Tukey's Multiple Comparison Test.

Examples of a cross-section through the center of the defect and a micro-CT derived outer view of mineralized tissue within a representative defect from each group are shown in Figure 1 (left column). The cross-sectional views show varying amounts of irregular radio-opacity in control, AOC- and ORC-treated defects respectively representing new bone formation. The MFC treated defects have clearly-defined margins indicating the absence of any new bone formation. These observations are reflected in the micro-CT derived 3-D reformulated images of mineralized tissue showing substantial, well developed mineralization in the control and AOC-treated defects, a more diffuse structure of mineralization in the ORC-treated defects and a virtual absence of mineralized tissue in the MFC-treated defects (Figure 1, center and right column). Micro-CT derived quantitative architectural parameters are shown in Table 1 and Figure 2. The tissue volume (TV) analyzed for each defect was comparable for all defect groups (Table 1). Normalization of the measured mineralized bone volume (BV) by TV, showed no difference in mineralization between AOC-treated and control defects (Figure 2) yielding fractional bone volume (BV/TV) in the following order Control = AOC > ORC >> MFC with BV/TV values of 0.40, 0.42, 0.25 and 0.05 respectively (Figure 2). Mineralization (Figure 2) in the AOC-treated and control defects was markedly higher than MFC-treated defects (8-fold difference, p <0.001) or ORC-treated defects (1.5 fold difference, p <0.05). ORC-treated defects showed more mineralization than MFC-treated defects (5 fold difference, p <0.01). The bone mineral density measured within the defects were comparable between all groups, indicative that the quality of new bone formed was consistent for all treatment groups (Table 1).

**Table 1:** Micro-CT derived architectural parameters for 2.7 mm circular defects at 17 days post-surgery.

MEASURE	CONTROL		AOC		ORC		MFC	
	Average	SEM	Average	SEM	Average	SEM	Average	SEM

**TV (mm^3^)**	5.10	0.18	5.34	0.22	4.77	0.10	5.18	0.14

**BV (mm^3^)**	2.19	0.34	2.13	0.17	1.21	0.16	0.27	0.06

**BMD (mgmL^-1^)**	696.6	10.7	665.6	11.4	637.1	8.8	669.2	12.2

Key: TV = volume of the whole defect; BV = mineralized tissue; BMD = density of mineralized tissue within the volume of interest.

**Figure 3:**
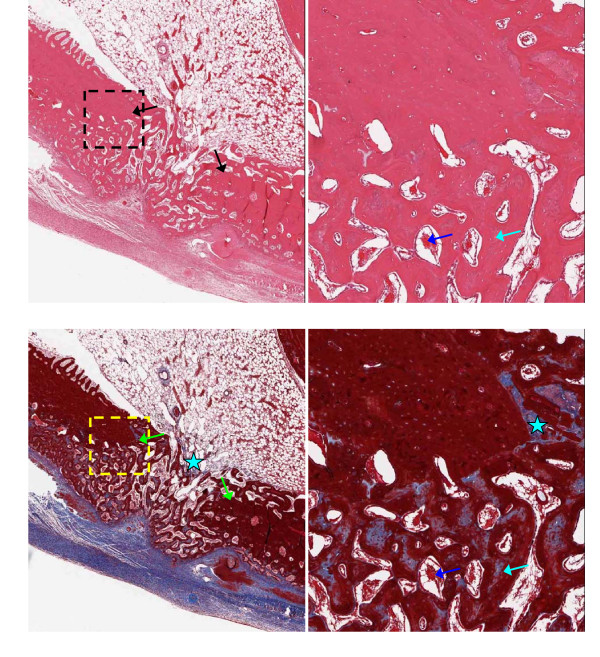
**Microscopy of control defects at 17 days post-surgery**. Untreated (Control) adjacent 5 μm demineralized bone sections stained with H&E (upper) and Trichrome-EVG at ×2 (left) and ×10 magnification. The edge of the defect is identified with black/green arrows, and the area of magnification shown with a dotted box. Defects show good healing with newly formed woven bone (light blue arrows) rich in cellular activity (osteoid shown with dark blue arrows). Blue stars indicate native collagen meshwork throughout the tissue.

**Figure 4:**
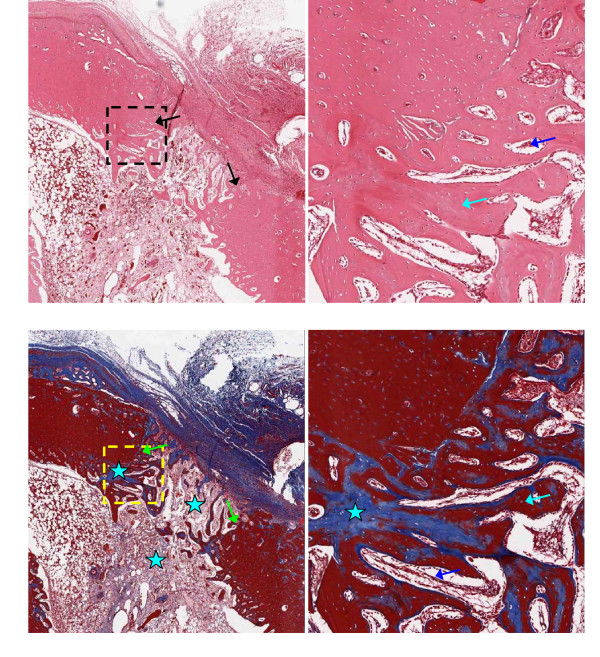
**Microscopy of alkylene oxide-treated defects at 17 days post-surgery**. Alkylene oxide copolymer-treated adjacent 5 μm demineralized bone sections stained with H&E (upper) and Trichrome-EVG at ×2 (left) and ×10 magnification. The edge of the defect is identified with black/green arrows, and the area of magnification shown with a dotted box. Defects show good healing with newly formed woven bone (light blue arrows) rich in cellular activity (osteoid shown with dark blue arrows). Blue stars indicate native collagen meshwork throughout the tissue.

**Figure 5:**
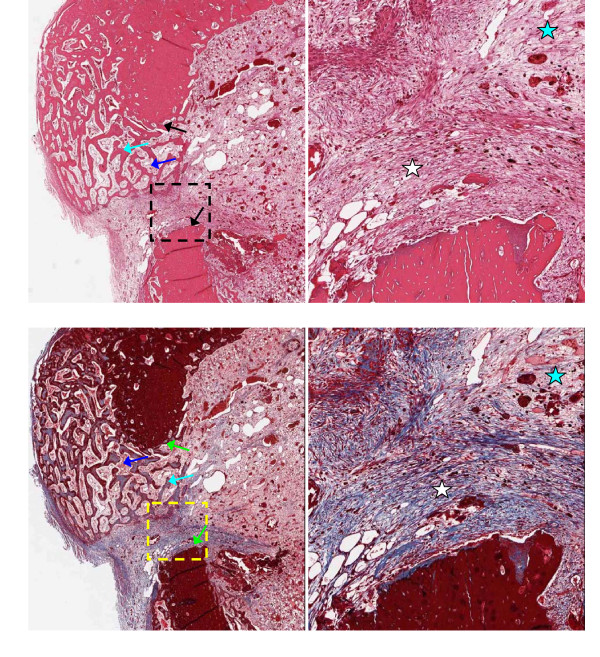
**Microscopy of oxidized regenerated cellulose-treated defects at 17 days post-surgery**. Oxidized regenerated cellulose-treated adjacent 5 μm demineralized bone sections stained with H&E (upper) and Trichrome-EVG at ×2 (left) and ×10 magnification. The edge of the defect is identified with black/green arrows, and the area of magnification shown with a dotted box. Defects show partial healing with newly formed woven bone (light blue arrows) rich in cellular activity (osteoid shown with dark blue arrows). Early fibrotic tissue is observed within the defect (white stars). Blue stars indicate native collagen meshwork within the marrow and defect.

**Figure 6:**
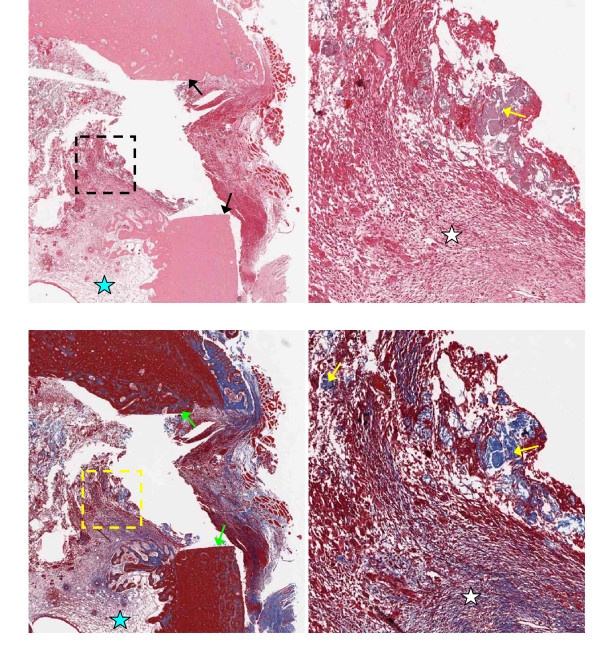
**Microscopy of microfibrillar collagen-treated defects at 17 days post-surgery**. Microfibrillar collagen-treated adjacent 5 μm demineralized bone sections stained with H&E (upper) and Trichrome-EVG at ×2 (left) and ×10 magnification. The edge of the defect is identified with black/green arrows, and the area of magnification shown with a dotted box. Defects show minimal healing with no evidence of newly formed woven bone. Dense bundles of residual collagen are observed (yellow arrows) and early fibrotic tissue within the marrow space (white stars). Blue stars indicate native collagen meshwork within the marrow space.

Representative 5 μm thick demineralized histology slices are shown in Figures 3, 4, 5 and 6, stained with H&E (upper panels) or trichrome-EVG at ×2 (left panels) and ×10 magnification. For untreated (control) and AOC-treated defects, much of the defect area is filled with newly formed woven bone rich in cellular activity, with no evidence of fibrotic scar tissue (Figures 3 and 4) nor residual polymer (Figure 4). Trichrome-EVG stained sections highlight Type 1 collagen within the areas of newly formed woven bone throughout the defect, indicative of good healing and an absence of fibrotic scar tissue (Figures 3 and 4). For ORC-treated defects (Figure 5), approximately half of the defect area is filled with newly formed woven bone and native collagen mesh, with the remaining portions showing early fibrotic tissue.

H&E and trichrome-EVG staining of MFC-treated defects (Figure 6) demonstrate negligible new bone formation with the margin of the defect clearly visible and evidence of early fibrotic tissue (dense collagen mesh) within the defect. The trichrome-EVG stain clearly demonstrates the presence of residual MFC hemostatic material within the marrow space of the tibia, seen as dense organized collagen bundles stained blue, and native collagen meshwork to a lesser extent as observed in the AOC-treated and untreated (control) defects (Figure 6).

## Discussion

### Model

Bone defects in animal studies that test efficacy of an agent intended to facilitate bony repair require a defect large enough to preclude spontaneous healing. In contrast, testing of hemostatic agents should be performed on defects smaller than a critical size, as such models demonstrate the effects of these agents on bone healing [18]. In our study, the 2.7 mm defect was non-critical, and could be created reproducibly with precision. The time point chosen reflects a point at which the defect would be largely healed on gross inspection but lacking complete mineralization within the defect, and at which micro-CT could detect the greatest differences between test groups. As a precise method to assess healing, micro-CT is an effective technique to quantitate mineralization within a defect as shown in calvarial bone defects in a rat model after application of bonewax or AOC compared to no hemostatic material [6]. Micro-CT derived volume fraction of bone provides an accurate measure of mineralized tissue within a 3-dimensional volume of interest as opposed to extrapolating values based on representative surface based (2-D) measurements of undemineralized histological sections [27]. For this study we chose a rabbit tibial defect as we were interested in using a long bone defect and a larger animal. The mineralized bone fraction can be directly correlated with torsional rigidity in a murine femur fracture model [29].

### Bone hemostasis

Both AOC and MFC achieved immediate and effective bone hemostasis. MFC required the use of an instrument for application to the defect whereas placement of AOC was simplest using a gloved finger. Oxidized regenerated cellulose achieved hemostasis 1-2 minutes after application. Effective hemostasis was maintained following application of all three hemostatic materials studied.

### Alkylene oxide copolymer

AOC is a hydrophilic waxy material that sticks well to wet surfaces, which makes it well-suited for bone hemostasis. Similar to bonewax, AOC achieves immediate hemostasis by tamponade and does not act biochemically [1,14,24], AOC polymer dissolves from the site of application within 48 hours [14,24] and is excreted unchanged primarily via renal filtration [25,26]. In this study, micro-CT analysis showed normal early bone healing in tibiae after the use of AOC, confirmed by histological analysis when compared to control defects where no hemostatic material was applied. This finding is consistent with earlier reports for the use of AOC for bone hemostasis [6-8]. Magyar et al. analyzed bone healing in a rat calvaria defect model using micro-CT, and found that AOC treated defects healed as well as, and possibly earlier than controls [6]. In a study employing a rabbit iliac crest defect model, the authors concluded that AOC does not interfere with bone healing at 6 weeks when evaluated using light microscopy [30]. A water-soluble AOC wax comprised solely of poloxamers (polyoxyethylene-polyoxypropylene-polyoxyethylene triblock copolymers) was an effective bone hemostasis material that was not evident at the implantation site at 72 hours, showed good healing as early as 10 days post-surgery and healing was comparable to untreated defects up to 42 days in a rat femur defect model as determined by X-ray and histological analyses [24].

### Oxidized regenerated cellulose

ORC relies upon multiple mechanisms of action for hemostasis, including physical and mechanical actions in tamponade, and surface interactions with proteins, platelets. ORC may also promote hemostasis chemically due to its low pH, which generates a brownish artificial clot containing acid hematin. ORC inhibited early bone healing in our study as compared to control and AOC (*P *< 0.05), but less so than MFC (*P *< 0.01). This finding is consistent with a number of previous animal studies [5,16,31]. Ibarrola et al. [5] examined bone healing in a rat tibial defect at multiple time points using light microscopy. ORC caused an intense inflammatory response and impaired osseous regeneration with residual material still present in the defects after 120 days [5]. These adverse effects were present to a lesser degree even when ORC was removed at the time of surgery [5]. Conversely, Finn et al., found no residual ORC two months after application in a canine iliac crest defect model and did not observe any adverse effects on bone regeneration on microscopic examination [3]. In a more recent study, Dias et al. employed histomorphological techniques to compare the effect of laboratory grade oxidized cellulose with a Type 1 bovine collagen sponge on bone healing in a 4 mm diameter bone defect sheep model. Although little difference was reported between the two groups, and bone healing was assessed as complete after 6 to 8 weeks, no control group was included in the study [15].

### Microfibrillar collagen

The classical powder form of MFC is a microcrystalline, water-insoluble, partial acid salt of collagen [17]. It is a dry, fluffy fleece that adheres well to bleeding surfaces and provides immediate hemostasis [1,18]. The hemostatic properties of MFC rely on the promotion of platelet aggregation as well as physically blocking bleeding vessels. Advantages of collagen fleece are: fast induction of hemostasis; low tissue reaction; and rapid resorption [16]. A major disadvantage of using collagen fleece is difficulty in manipulating the agent when attempting to apply it to the area of bleeding [1]. MFC is actively degraded *in vivo *and is reported to be removed from the site of application in 45 to 90 days [1,3,21]. The surprising finding of our study is the almost complete absence of bone healing 17 days following application of MFC, an effect similar to that observed with the use of nonresorbable bone wax [6]. The histological images for MFC-treated defects show a clearly defined defect margin indicating that minimal healing occurred over the 17 post-surgical days. These findings are consistent with histological evaluation of rabbit cranial defects filled with MFC that were shown to be significantly larger than untreated control defects at 4 and 7 weeks post-surgery [32].

In contrast, other authors have found little or no adverse effect of MFC on bone healing at time points in excess of 2 months based on qualitative observations using histological techniques [3,17,33]. Cobden et al. performed osteotomies of the greater trochanter in a canine model and found no evidence that MFC interfered with bone healing at three months [17]. In a study that employed a 5 mm rat tibial defect, residual MFC material was found in the defects at 90 days, although the authors concluded MFC did not impede bone healing since the bone defects had healed at 60 days [33]. In a subsequent study, Finn et al. examined the effects MFC on osseous regeneration in a canine iliac crest defect model. On qualitative microscopic examination after 2 months residual MFC was observed in the defects, but no adverse effects on bone regeneration was noted [3].

Our study investigated the effect of MFC on early bone healing at 17 days post-surgery, which may explain the discrepancy between our findings and those of other authors at much longer post-surgical time points [3,17,33]. Although effective at achieving immediate and effective hemostasis, the adverse effect of MFC on osteogenesis would suggest that this agent would impair healing in the clinical setting, and this finding is in agreement with the recommendation that MFC should be removed from the site of application as it interferes with bone healing [16].

## Conclusion

Microfibrillar collagen and alkylene oxide copolymer achieved immediate and effective bone hemostasis. Oxidized regenerated cellulose achieved hemostasis 1-2 minutes after application. Effective hemostasis was maintained following application of all three hemostatic materials studied. In our model, early bone healing was significantly impaired by the presence of microfibrillar collagen and impeded by the presence of oxidized regenerated cellulose. Alkylene oxide copolymer did not inhibit bone healing when compared to untreated (control) defects and thus may be a good clinical agent in cases where bony fusion is critical and where immediate hemostasis is required.

Our results confirm the use of alkylene oxide copolymer does result in faster bone healing compared to either oxidized regenerated cellulose or microfibrillar collagen, presumably because AOC is cleared from the bone defect much earlier.

## Competing interests

JKA and TCF are employees of Ceremed Inc.

JKA and TCF are co-inventors of Ostene^® ^and receive a royalty payment

BH is a paid consultant for Ceremed, Inc.

CEM is married to JKA who is an employee of Ceremed Inc.

Ceremed Inc., supported the work, provided the test materials used for this study and is financing the article processing charge.

## Authors' contributions

JKA and TCF designed the study and drafted the manuscript. BH, KK and ZY carried out the operation and clinical examination of the animals. ST and EA carried out the micro-CT acquisition and data analysis. SMD and CEM carried out the histological imaging and analysis, and CEM performed statistical analyses on all data. All authors read and approved the final manuscript.

## Pre-publication history

The pre-publication history for this paper can be accessed here:

http://www.biomedcentral.com/1471-2482/10/37/prepub
